# Sural artery perforator flap with posterior tibial neurovascular decompression for recurrent foot ulcer in leprosy patients

**DOI:** 10.3205/iprs000103

**Published:** 2017-01-31

**Authors:** Hossam El-din Ali Ismail, Mohamed Hassan El Fahar

**Affiliations:** 1 Plastic Surgery Department, Mansoura University, Mansoura, Egypt

**Keywords:** recurrent neuropathic heel ulcer in leprosy, reverse sural fasciocutaneous flap with neurovascular decompression

## Abstract

**Introduction:** The sensory loss and alteration of the shape of the foot make the foot liable to trauma and pressure, and subsequently cause more callus formation, blisters, and ulcers. Foot ulcers usually are liable to secondary infection as cellulitis or osteomyelitis, and may result in amputations. Foot ulcers are a major problem and a major cause of handicaps in leprosy patients. The current study is to present our clinical experience and evaluate the use of sural flap with posterior tibial neurovascular decompression (PTND) in recurrent foot ulcers in leprosy patients.

**Patient and methods:** A total number of 9 patients were suffering from chronic sequelae of leprosy as recurrent foot ulcers. All the patients were reconstructed with the reverse sural artery fasciocutaneous flap with posterior tibial neurovascular decompression from September 2012 to August 2015. Six patients were male and three were female with a mean age of 39.8 years (range, 30–50 years). All the soft tissue defects were in the weight-bearing area of the inside of the foot. The flap sizes ranged from 15/4 to 18/6 cm. Mean follow-up period was 21.2 months (range, 35–2 months).

**Results:** All the flaps healed uneventfully. There was no major complication as total flap necrosis. Only minor complications occurred which were treated without surgical intervention except in two patients who developed superficial necrosis of the skin paddle. Surgical debridement was done one week later. The flap was completely viable after surgery, and the contour of the foot was restored. We found that an improvement of sensation occurred in those patients in whom the anesthesia started one year ago or less and no sensory recovery in patient in whom the anesthesia had lasted for more than two years.

**Conclusion:** The reverse sural artery flap with posterior tibial neurovascular decompression provides a reliable method for recurrent foot soft tissue reconstruction in leprosy patients with encouraging function and aesthetic outcomes. It is a quick and easy procedure.

## Introduction

Although the prevalence of leprosy has decreased, foot affection due to neuropathy is common in leprosy patients [1]. Neuropathic affection can lead to skin fissures, contractures and/or clawing of the toes, and irreversable neuroosteoarthropathy [2]. The sensory loss and affected shape of the foot make the foot liable to trauma and pressure, and subsequently cause more callus formation, blisters and ulcers. Foot ulcers are usually liable to secondary infection as cellulitis or osteomyelitis, and may result in amputations. Foot ulcers are a major problem and considered as a major cause of handicaps in leprosy patients [3]. 

The majority of neuropathic ulcers occur on the plantar surface of the feet, with about 70% on the forefoot [4] with high recurrence rate [5]. Some authors thought that these ulcers can be prevented by the use of suitable footwear and preventive foot care [6], [7]. 

Different studies have mentioned the posterior tibial neurovascular decompression (PTND) for the management of plantar ulcers in leprosy patients. Carayon and Bourrel predict the importance of changes in the posterior tibial nerve (PTN) and vessels due to its compression in the tarsal tunnel [8], [9], also Palande detect the importance of ischemia changes and PTND for treatment of plantar ulcers in leprosy patients [10]. Although different authors mentioned PTND for the treatment of the neuropathic heel ulcers, no one was using it with reverse sural artery flap as a resurfacing procedure for the management of recurrent ulcers in the foot of leprosy patients. 

In the current study, we present our clinical experience with the utilization of PTND with reverse sural artery fasciocutaneous perforator flap coverage in recurrent foot ulcers in leprosy patients.

## Material and methods

### Patient demographic data

A total number of 9 patients, 6 males and 3 females, were suffering from recurrent heel ulcers as chronic sequelae of leprosy (Table 1 (Tab. 1)). All the patients received the antileprosy treatment and became non-infective. All the patients were reconstructed with the reverse sural artery fasciocutaneous perforator flap (RSAF) with PTND at the period from September 2012 to October 2015. The mean age of the patients was 39.8 years (range, 30–50 years). All the soft tissue defects were in the pressure areas of the foot. The size of the flap varied from 15/4 to 18/6 cm (Table 1 (Tab. 1)). The entire patient was subjected to plain X-ray to exclude calcaneal osteomyelitis. The following absolute and relative exclusion criteria were applied; the absolute criteria include: patients not fit for surgery, patient with vascular disorders, peroneal artery perforators not detected and non ambulant. The relative criteria include smokers who had to stop smoking at least one month before procedure and diabetic patients who had to have good diabetic control before the procedure. Preoperative detection of the site of the peroneal artery perforators by using the doppler is essential.

### Operative procedure

**Figure 1:**
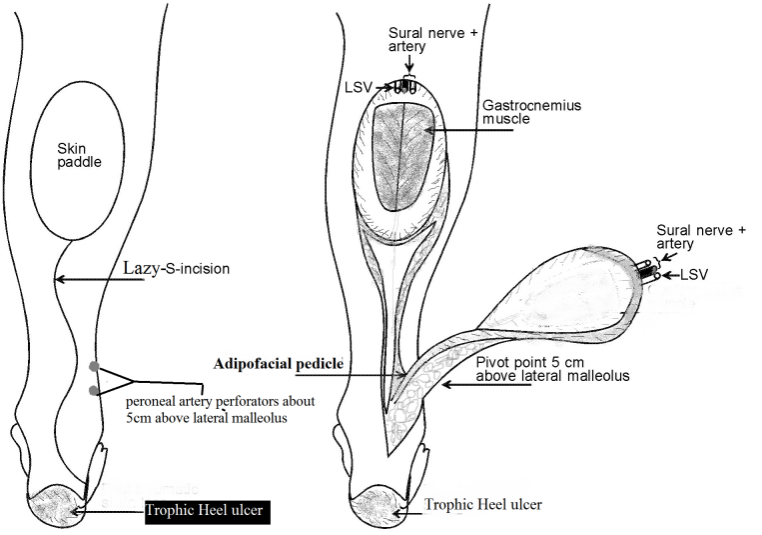
Schematic drawing of distally based fasciocutaneous flap with adipofascial pedicle

The operative technique is well described in previous publications [11], [12], [13] and will be briefly mentioned here (Figure 1 (Fig. 1)). 

The operations were performed under spinal anesthesia, then the patients were positioned prone on the operating table and the tourniquet was used. The wound was debrided and copiously irrigated with saline 0.9%. Preoperative marking of the axis of the flap which represents the course of the medial sural nerve was done.

**Figure 2:**
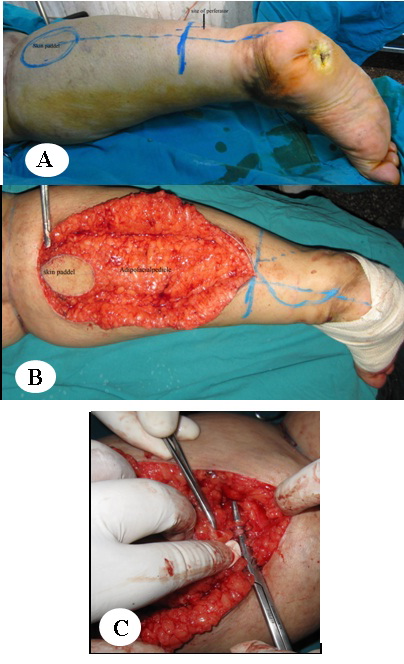
43-year-old female patient with recurrent neuropathic heel ulcer due to leprosy for 2 years. A: Marking of the flap. B: Intraoperatively, exposure of the pedicle (sural nerve, sural vessels, and LSV). C: Intraoperatively, during exposure of the neurovascular pedicle.

The skin paddle of the fasciocutanous flap was outlined according to the size of the defect. The pivot point is about 5 cm above the lateral malleolus, the location of the lowermost perforator from the peroneal artery. At the proximal end of the flap, small saphenous vein (SSV), the median superficial sural artery (MSSA), and the sural nerve (SN) were ligated and divided. The pedicle which contains the neurovascular bundle was exposed through lazy-S-skin incision and raised with a small cuff of fascia around it up to the level of the outlined flap (Figure 2 (Fig. 2)). The proximal and lateral margins of the flap were incised through the skin and deep fascia. The deep fascia was sutured to the skin island by tacking suture and dissected from the muscles, provided that the vessels and the nerve are included in the flap. The pedicle was dissected to the level about the lowermost peroneal perforator in the posterolateral intermuscular septum. The flap was transferred to the defect either by incising the skin bridge or above the skin (one flap). The flap donor site can be closed primarily if the skin paddles are less than 3 cm wide after wide undermining. Otherwise, a skin graft can be applied. The tourniquet was then released to achieve haemostasis and testing of the good vascularity of the flap was done before insetting the flap. The flap was then inset, the deep fascia of the flap sutured to fascia and a drain was introduced underneath the flaps. Surgical decompression of the entrapped posterior tibial nerve at the tarsal tunnel was done through the lowermost part of the incision above the medial malleolus for all patients. Also neurolysis was done in 6 cases.

Postoperatively the patients continue to receive IV antibiotic for 7–10 days. The flap was checked for viability after 24 hours. The drain was removed after 84 hours. All patients were kept in bed for 1 week. The patients were discharged on day 10 to 14 postoperatively after good take of the skin graft and followed up at regular intervals. The average follow period was 21.2 months (ranging from 35 to 2 months). None of the patients had problems in wearing medical shoes with silicone heel pad.

## Results

Nine adult patients with recurrent ulcers were included in the study. All the patients underwent single stage soft tissue reconstruction using a reverse sural artery fasciocutaneous flap with PTND except one case in which separation of the pedicle was done after 3 weeks postoperatively.

**Figure 3:**
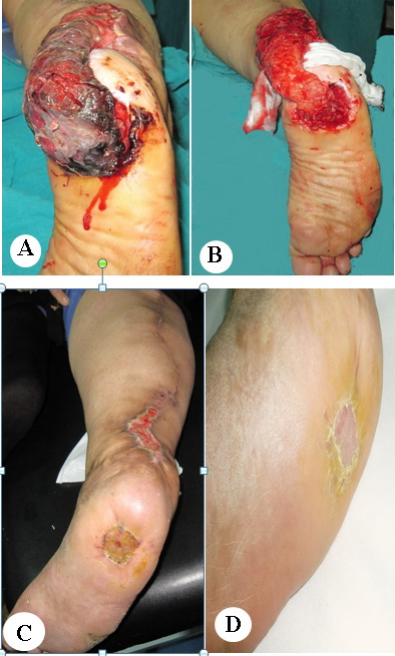
Patient from Fig. 2. A: Postoperative congestion and superficial necrosis of the sural flap after one week. B: After debridement, one week postoperatively. C: After separation of the flap and healing of the split-thickness skin graft. D: 7 months postoperatively.

All the flaps were successful with minor complications. Congestion occurred for 72 h postoperatively in three flaps, and then improved. Superficial necrosis of the skin paddle occurred in two flaps treated by surgical debridement two weeks later (Figure 3 (Fig. 3)). Other complications were a delayed take of the partial thickness graft and hypertrophic scars at the edge of the skin graft in donor and recipient site.

The mean operative time was 95 minutes (range, 80 to 120 minutes). The mean time of hospital stay was about 8.9 days (range, 7–12 days) (Table 2 (Tab. 2)). 

We observed that good improvement of sensation in the foot occurred in the patients in whom anesthesia had started one year ago or less and no sensory recovery in patients in whom the anesthesia had lasted for more than two years. Healing was uneventful and function satisfaction of the patients about 70%, but aesthetic satisfaction about 80% (Table 2 (Tab. 2)).

## Discussion

Plantar ulcers are quite common in leprosy. The treatment of such ulcers is different from that of traumatic plantar ulcers. The affection of the PTN is common in leprosy, and subsequently sensory loss of the foot with paralysis of the small muscle of the foot occurres. Unfortunatly foot ulcers are complications caused by the loss of sensation of the foot. Theoretically, the recurrence of these ulcers can be prevented if sensation is restored in the foot. Many authors have used PTND for the treatment of foot ulcers. Carayon and Bourrel detected the importance of changes in the PTN and vessels due to its compression in the tarsal tunnel [8], [9] also Palande detected the importance of ischemia and decompression of the PTN for management of plantar ulcers [10].

Although different authors mentioned the PTND for the treatment of the neuropathic heel ulcers, no one used it with reverse sural artery flap as a resurfacing procedure for the treatment of recurrence of foot ulcers in leprosy patients. 

Prakash et al. were the first to describe the use of PTND with various reconstructive techniques in sixteen patients. They found a good sensory improvement in 6 patients, poor sensory improvement in another 6 patients and no sensory improvement in 4 patients. They found also marked improvement of sensation occurring in those patients in whom the sensory affection had lasted one year or less and no sensory recovery in patients in whom the anesthesia had lasted more than two years [14].

In the current study 9 leprosy patients, 6 males and 3 females, were subjected to reconstruction of recurrent foot ulcers using a reverse sural artery fasciocutaneous flap with peroneal artery perforators. All the flaps were successful with minor complications as congestion for 72 h of one flap, superficial necrosis of the skin paddle, delayed take of the partial thickness graft, and hypertrophic scars at the edge of the skin graft in donor and recipient site. We found good improvement of sensation in patients with a history of anesthesia for one year or less and no sensory recovery in patients with a history of anesthesia for more than two years. This results coincide more or less with Prakash et al. in their series [14].

Healing was uneventful, and all the patients were highly satisfied with the outcome. The average follow-up period was 21.2 months (ranging from 35 to 2 months). The patients had no problems in wearing medical shoes with silicone heel pad. 

The reverse sural artery flap has a reverse venous drainage, so venous congestion is commonly occurring. Several authors tried to solve this problem by the use of leeches [15], of an angiocatheter inserted into the cut end of the short saphenous vein to drain blood from the congested flap [16], or by using the “venous supercharging” principle (end-to-end anastomosis between the cut end of the short saphenous vein and any superficial vein) [17]. Furthermore, others tried to enhance both the arterial supply and the venous drainage by delaying the flap [18], [19], [20], [21]. 

In 2001, Al-Qattan [22] recommended raising the reverse sural artery flap to include gastrocnemius muscle cuff around the sural pedicle to maintain a “mesenteric” connection between the flap and the sural pedicle. He observed an improvement in both the arterial supply and the venous drainage.

## Conclusion

The reverse sural artery flap with PTND provides a reliable method for recurrent foot soft tissue reconstruction in leprosy patients with encouraging function and aesthetic outcomes. It is a quick and easy procedure.

## Notes

### Competing interests

The authors declare that they have no competing interests.

## Figures and Tables

**Table 1:**
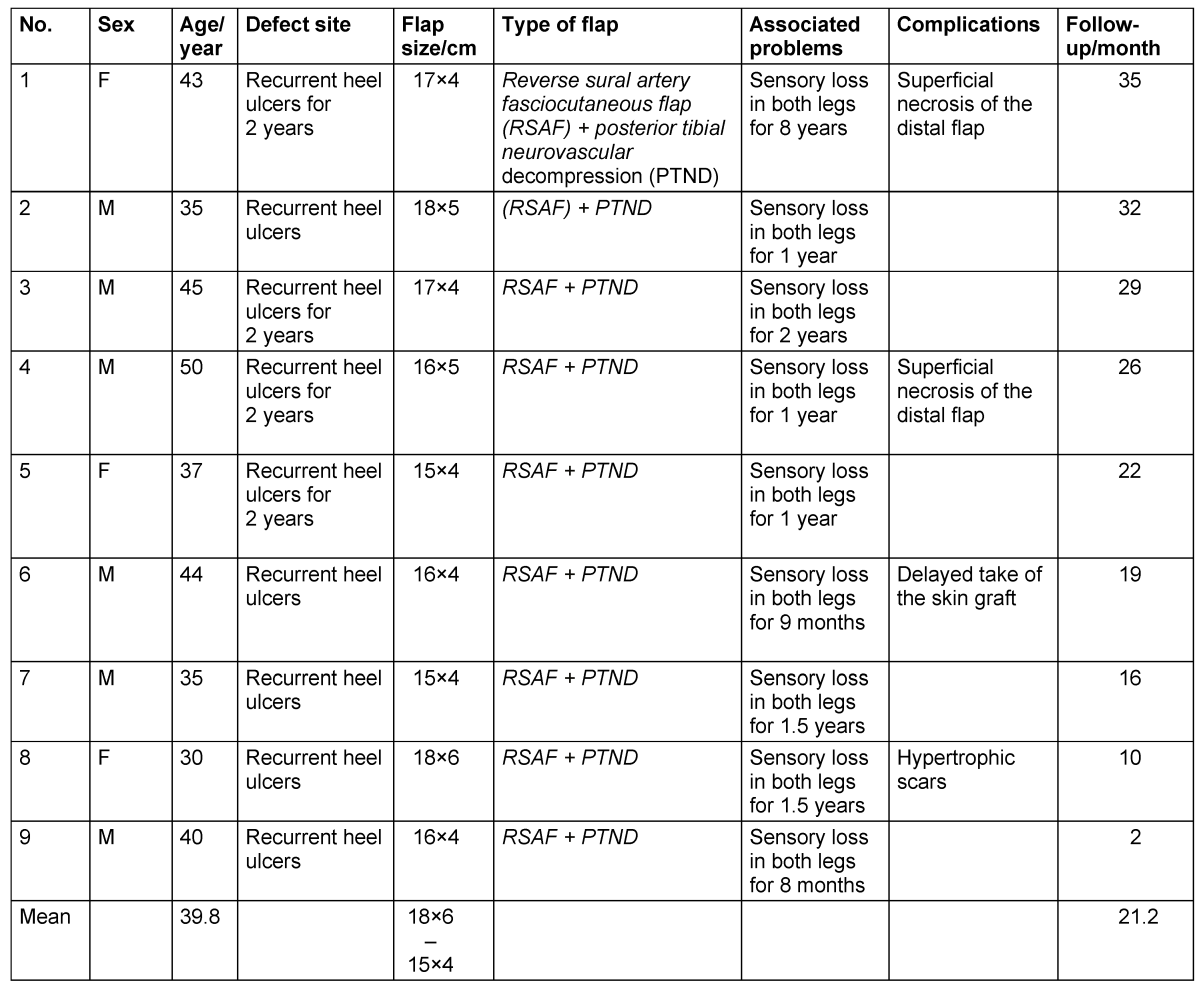
Summary of patients’ data

**Table 2:**
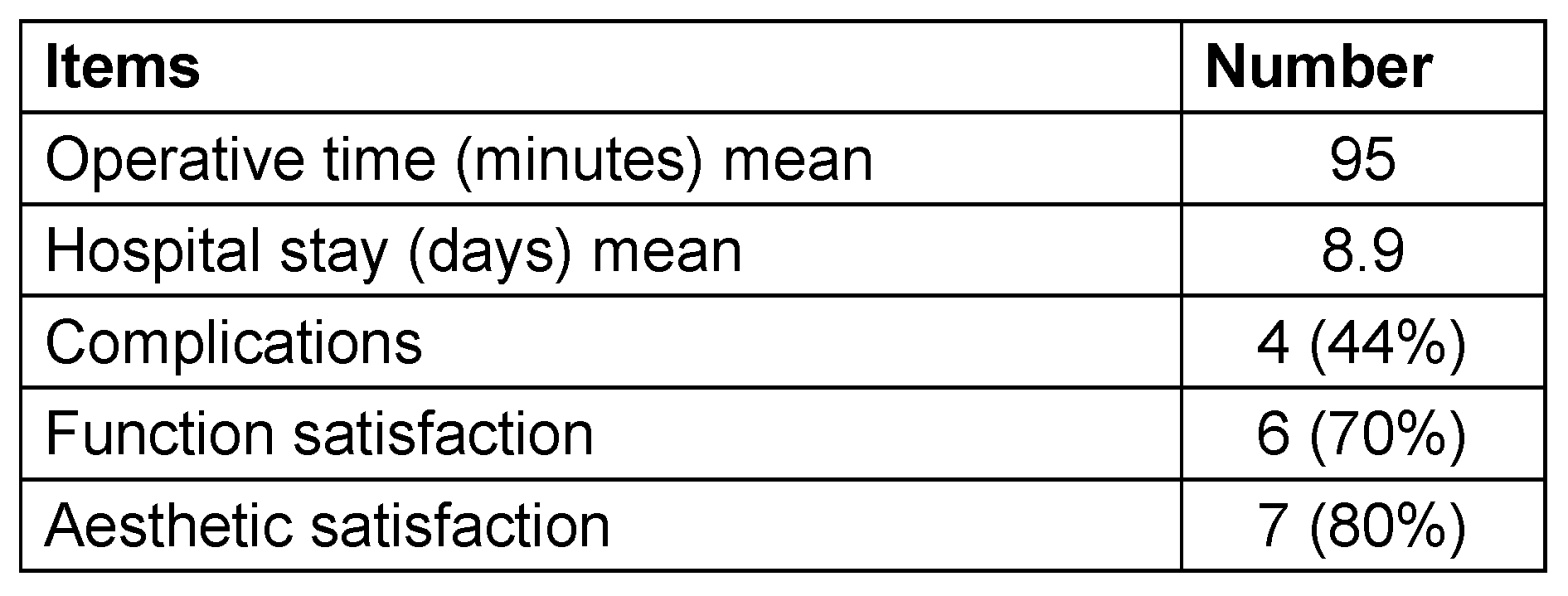
Outcome data
